# Secondary mania related to acquired immunodeficiency syndrome (AIDS). Case report

**DOI:** 10.1192/j.eurpsy.2023.1454

**Published:** 2023-07-19

**Authors:** D. C. Gliția, R. Cozma, C. A. Crișan

**Affiliations:** 1 Psychiatry I, Infectious Diseases Clinical Hospital Cluj-Napoca; 2 Psychiatry I, Emergency County Hospital Cluj-Napoca; 3 Department of Neurosciences, Discipline of Psychiatry and Pediatric Psychiatry, Iuliu Hațieganu University of Medicine and Pharmacy Cluj-Napoca, Cluj-Napoca, Romania

## Abstract

**Introduction:**

Neuropsychiatric manifestations in human immunodeficiency virus (HIV) infection are uncommon but salient once they emerge to the surface. These symptoms can be the result of direct or indirect effects of the virus on the central nervous system (CNS). In particular, HIV related mania can complicate any stage of the infection but increases its frequency with the progression of HIV infection to the final stage.

**Objectives:**

The objective of this case report is to rase awareness about secondary mania due to HIV infection and the importance of etiological treatment in mental disorders.

**Methods:**

We herein report the case of a 27-year-old, male patient, who was admitted to our Psychiatric Clinic I Cluj-Napoca, with a 3-week history of typical manic symptoms such as: elated mood, alternating with episodes of irritability, talking too much, familiarity, multiple future plans, hypersexuality, social disinhibition and decreased need for sleep. Throughout the hospitalization, the course of the manic symptomatology did not improve, additionally the patient started to exhibit neurological symptoms accompanied by complex visual hallucinations. Prior to this episode he reported depressive symptoms, predominantly anhedonia, apathy, and social withdrawal but without meeting the clinical severity threshold. The patient had no family history of a mental disorder. A psychopharmacological treatment was initiated (atypical antipsychotic Quetiapine XR 300 mg/day initially, and then switched to Olanzapine 10 mg/day, mood stabilizer Valproic Acid 1,5 g/day), but he developed significant extrapyramidal side effects.

**Results:**

Blood tests revealed: leukopenia, lymphopenia, thrombocytopenia, subsequently hepatic cytolysis, and high CRP. Psychometric evaluation revealed: Young Mania Rating Scale (YMRS) score 33/60 – moderate mania, Positive and Negative Syndrome Scale (PANSS)- total score 51 (16/49 Positive; 7/49 Negative; 28/112 General Psychopathology). MRI: T2 and FLAIR hyperintense extended areas in the bilateral periventricular white matter and in the internal capsule. The anamnesis, heteroanamnesis, paraclinical investigations led us to a diagnosis of secondary mania related to HIV infection. The patient was transferred to the Infectious Diseases Clinical Hospital for a targeted antiretroviral therapy (Raltegravir 800 mg/day, Emtricitabine/Tenofovir disoproxil 200mg/245 mg).

**Conclusions:**

Recognizing and controlling HIV secondary mania should be of high importance given its association with heightened sexual behavior and substance abuse which can result in an elevated risk of transmitting the infection to other people.

**Disclosure of Interest:**

None Declared

